# Increased Risk of Dementia in Patients with Tension-Type Headache: A Nationwide Retrospective Population-Based Cohort Study

**DOI:** 10.1371/journal.pone.0156097

**Published:** 2016-06-07

**Authors:** Fu-Chi Yang, Te-Yu Lin, Hsuan-Ju Chen, Jiunn-Tay Lee, Chun-Chieh Lin, Chia-Hung Kao

**Affiliations:** 1 Department of Neurology, Tri-Service General Hospital, National Defense Medical Center, Taipei, Taiwan; 2 Division of Infectious Diseases and Tropical Medicine, Department of Internal Medicine, Tri-Service General Hospital, National Defense Medical Center, Taipei, Taiwan; 3 Management Office for Health Data, China Medical University Hospital, Taichung, Taiwan; 4 College of Medicine, China Medical University, Taichung, Taiwan; 5 Graduate Institute of Clinical Medical Science and School of Medicine, College of Medicine, China Medical University, Taichung, Taiwan; 6 Department of Nuclear Medicine and PET Center, China Medical University Hospital, Taichung, Taiwan; National Health Research Institutes, TAIWAN

## Abstract

**Purpose:**

The association between primary headaches, including tension-type headache (TTH) as one of the most common primary headache disorders, and dementia remains controversial. In this nationwide, population-based, retrospective, cohort study, we explored the potential association between TTH and dementia and examined sex, age, and comorbidities as risk factors for dementia.

**Methods:**

Using the Taiwan National Health Insurance Research Database (NHIRD) claims data, the sample included 13908 subjects aged ≥20 years with newly-diagnosed TTH in 2000–2006. The non-TTH group included 55632 randomly selected sex- and age-matched TTH-free individuals. All subjects were followed until dementia diagnosis, death, or the end of 2011. Patients with dementia, including vascular and non-vascular (including Alzheimer’s) subtypes, were identified using International Classification of Diseases Ninth Revision, Clinical Modification codes. Multivariate Cox proportional hazards regression models were used to assess the risk of dementia and dementia-associated risk factors, such as migraine and other medical comorbidities.

**Results:**

During the average follow-up of 8.14 years, the incidence density rates of dementia were 5.30 and 3.68/1,000 person-years in the TTH and non-TTH groups, respectively. Compared with the non-TTH group, the risks of dementia were 1.25 (95% confidence interval [CI], 1.11–1.42) and 1.13 (95% CI, 1.01–1.27) times higher in the women and >65-year-old TTH group, respectively. TTH patients with comorbidities had a higher risk of dementia. TTH patients had a greater risk of non-vascular dementia (hazard ratio, 1.21; 95% CI, 1.09–1.34) than the non-TTH group.

**Conclusion:**

TTH patients have a future risk of dementia, indicating a potentially linked disease pathophysiology that warrants further study. The association between TTH and dementia is greater in women, older adults, and with comorbidities. Clinicians should be aware of potential dementia comorbidity in TTH patients.

## Introduction

Tension-type headache (TTH) is the most common type of primary headache, typically presenting as a bilateral, non-throbbing headache with mild to moderate intensity. It is usually precipitated by stress and mental tension and does not worsen with routine physical activity [1]. Although the intensity of TTH is generally less severe than that of migraine, it is a significant condition owing to its high prevalence in the general population (lifetime prevalence up to 78%) [2]. Moreover, recent accumulating evidence suggests a relationship between primary headaches and cognitive status and dementia; however, most studies focused on migraine [3–10].

Dementia is one of the most common neurodegenerative diseases, with a significant impact on quality of life and public health [11]. It is characterized by slow progressive impairment of memory and at least one additional cognitive domain. Approximately 24.3 million people worldwide have dementia, and this is expected to increase to 81.1 million people by 2040 [12]. There are several types of dementia, with non-vascular dementia (non-VD) (including Alzheimer's disease [AD]) as the most common type, accounting for 80–90% of cases, followed by VD, which accounts for 10–20% of cases [13,14]. The risk of dementia tends to increase with aging, and other risk factors for dementia include a variety of medical conditions or comorbidities, such as diabetes, hypertension, dyslipidemia, ischemic heart disease (IHD), heart failure (HF), chronic obstructive pulmonary disease (COPD), depression, head injury, stroke, and Parkinson’s disease [15–19].

The relationship between somatic symptoms, such as headache, and the clinical presentation of dementia has been considered more than a simple coincidence [20], and previous registry and population-based cohort studies have suggested an association between migraine and the occurrence of dementia [5,21,22]. However, the results from other longitudinal studies of migraine and cognitive decline were mixed and somewhat controversial, with some of these studies failing to find an increased risk of cognitive decline with migraines [3–10]. Furthermore, the association between non-migrainous headache and dementia has rarely been studied, with only two population-based studies in Norway suggesting a potential association between non-migrainous headache and dementia [3,22]. In addition, although a previous validation study reported that 80% of individuals with non-migrainous headache might have TTH [23], differentiation between the effect of TTH, which is the most prevalent type of primary headache, and other-non-migrainous headaches is lacking. Although an association between migraine and non-VD (e.g., dementia with Lewy bodies) was reported in a dementia registry study in Sweden [5], the relationship between TTH and non-VD has not been investigated previously.

Based on the collective evidence, the association between primary headaches and dementia remains controversial, and the temporal relationship between TTH and dementia is unknown. Therefore, this nationwide, population-based cohort study aimed to explore the possible temporal association between TTH and dementia.

## Methods

### Data Source

Instituted in 1995, The National Health Insurance (NHI) system is a mandatory universal program that offers comprehensive medical care coverage to all Taiwanese residents. Our study used the Longitudinal Health Insurance Database 2000 (LHID 2000), a subset of the National Health Insurance Research Database (NHIRD). The LHID 2000 consists of historical claims data for one million patients randomly sampled from the entire insured population in 1996–2011. According to the government document, there was no significant difference in the distribution of sex and age between the insured people in the LHID 2000 and the original Taiwan NHI. The LHID2000 contains comprehensive information, including demographic, clinical visits, prescription details, and diagnostic codes based on the International Classification of Diseases, Ninth revision, Clinical Modification (ICD-9-CM). For security and privacy purposes, patient identity data is scrambled cryptographically by the NHIRD before being released for research. This study was exempted by the Institutional Review Board of China Medical University in central Taiwan (CMUH104-REC2-115).

### Study Population

The population-based cohort study aimed to investigate the association of TTH and the risk of dementia that included two groups: TTH group and non-TTH group. We identified patients who were 20 years of age and older with newly diagnosed TTH (ICD-9-CM 307.81 and 339.1) based on outpatient and/or inpatient care data in the NHIRD between 1 January 2000 and 31 December 2006 as the TTH group, and the diagnosed date of TTH was considered as the index date. For each patient with TTH, four insured subjects were selected from subjects without a diagnosis of TTH in the LHID 2000 as the non-TTH group and were frequency-matched by sex, age (every 5-years span) and year of index date. Both groups with a prior diagnosis of dementia (ICD-9-CM 290, 294.1, and 331.0) or with missing information on age and/or sex were excluded from this study.

### Covariates and Outcome

The demographic factors included sex and age (in age group of 20–44, 45–64, and ≥ 65 years). We considered diabetes (ICD-9-CM 250), dyslipidemia (ICD-9-CM 272), hypertension (ICD-9-CM 401–405), ischemic heart disease (IHD; ICD-9-CM 410–414), atrial fibrillation (AF; ICD-9-CM 427.31), heart failure (HF; ICD-9-CM 428), stroke (ICD-9-CM 430–438), depression (ICD-9-CM code 296.2, 296.3, 300.4, and 311), head injury (ICD-9-CM 310.2, 800, 801, 803, 804, 850–854, and 959.01), Parkinson’s disease (ICD-9-CM 332), migraine (ICD-9-CM 346), and chronic obstructive pulmonary disease (COPD; ICD-9-CM 491, 492 and 496), which were identified as comorbidities before the index date.

In this study, the primary outcome was dementia (ICD-9-CM 290, 294.1, and 331.0). Both TTH and non-TTH groups were observed from the index date to the diagnosed date of dementia, withdrawal from the NHI program, or the end of 2011, whichever occurred first. Furthermore, we examined different subtypes of dementia. VD was identified in the claims data using the International Classification of Diseases, Ninth Revision, Clinical Modification (ICD9-CM) code 290.4; all of the remaining patients with dementia were classified with non-VD, and patients with AD were differentiated from those with non-VD using ICD9-CM code 331.0.

### Ethics Statement

The NHIRD encrypts patient personal information to protect privacy and provides researchers with anonymous identification numbers associated with relevant claims information, including sex, date of birth, medical services received, and prescriptions. Therefore, patient consent is not required to access the NHIRD. This study was approved to fulfill the condition for exemption by the Institutional Review Board (IRB) of China Medical University (CMUH104-REC2-115). The IRB also specifically waived the consent requirement.

### Data Availability Statement

All data and related metadata were deposited in an appropriate public repository. The data on the study population that were obtained from the NHIRD (http://w3.nhri.org.tw/nhird//date_01.html) are maintained in the NHIRD (http://nhird.nhri.org.tw/). The NHRI is a nonprofit foundation established by the government.

### Statistical Analysis

The continuous variables were expressed by means and standard deviations (SD), whereas categorical variables were expressed by the numbers and percentages. Differences were examined using the Pearson's chi-squared test for categorical variables and the Student’s t test for continuous variables. The sex-specific, age-specific, and comorbidity-specific incidence density rates (per 1000 person-years) of dementia was calculated by using the number of newly diagnosed dementia dividing by person-years at risk in both groups. The cumulative incidence curves of dementia in the TTH and non-TTH groups were estimated using the Kaplan-Meier analysis and the difference between groups was compared using the log-rank test. We used the Cox proportional hazards models to assess the independent effects of TTH by adjusting for sex, age, and comorbidity in the multivariate model. We also perform sex-, age-, and comorbidity-stratified analyses to estimate the associated between TTH and the risk of dementia zoster. Finally, we examined the association between TTH and the risk of different subtypes of dementia. Hazard ratios (HRs) and 95% confidence intervals (CIs) were calculated to quantify the risk of dementia. We used SAS software (version 9.4; SAS Institute Inc., Cary, N.C., USA) for all statistical analyses. All statistical tests adopted a two-tailed significance level of 0.05.

## Results

**Fig 1:**
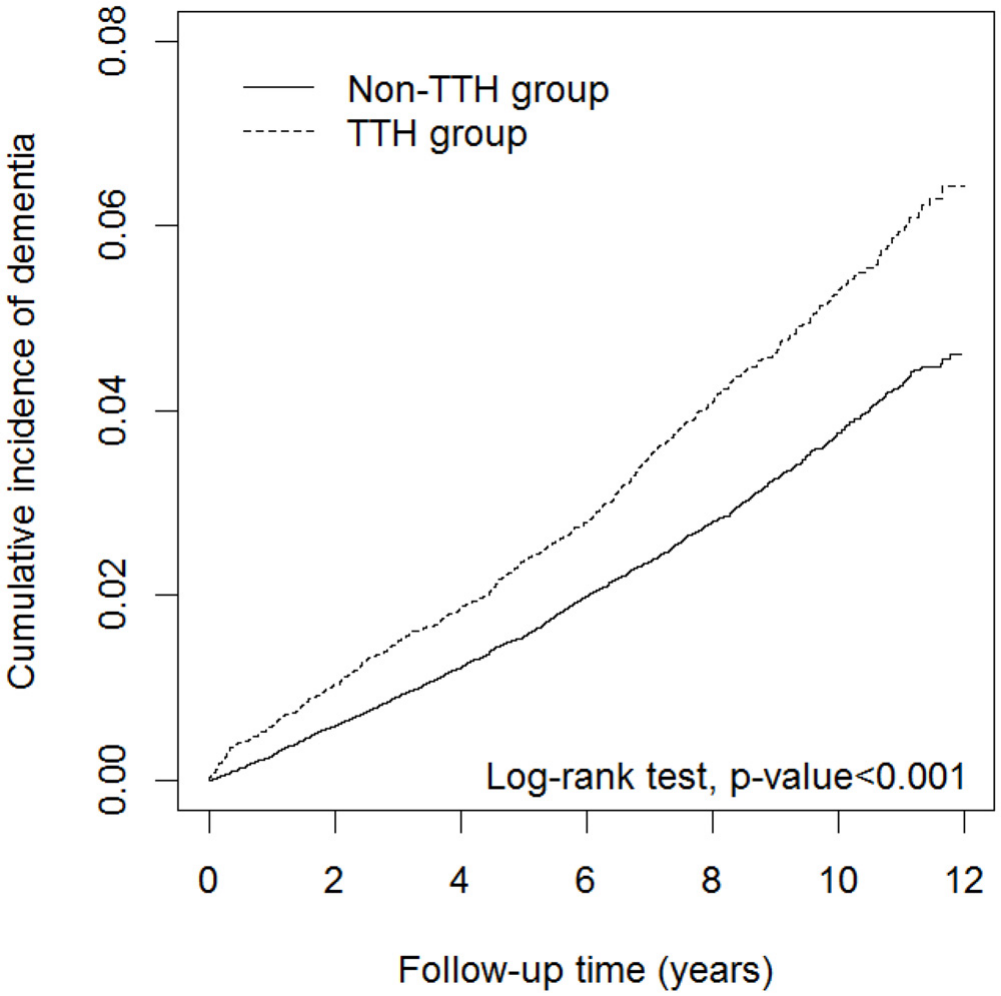
Cumulative incidence curves of dementia for groups with and without tension-type headache.

From 2000–2006, this study identified 13908 patients newly diagnosed with TTH (mean age = 49.1 years, SD = 15.4) and 55632 subjects without TTH (mean age = 48.8 years, SD = 15.6 years). There were no significant differences between the TTH group and non-TTH group in the distributions of sex and age. However, several comorbidities were significantly more prevalent among the TTH group than the non-TTH group; these included diabetes, dyslipidemia, hypertension, IHD, HF, stroke, depression, head injury, Parkinson’s disease, migraine, and COPD (p-value < 0.001) (Table 1). During an average follow-up of 8.14 years, a total of 604 (4.34%) patients of the TTH group and 1633 (2.94%) patients of the non-TTH group developed dementia. The result of Kaplan-Meier analysis of the cumulative incidence of dementia shows it to be significantly higher for patients with TTH than subjects without TTH (log rank test, p-value < 0.001), as shown in Fig 1. The incidence density rates of dementia was higher in the TTH group than in the non-TTH group (5.30 and 3.68 per 1000 person-years, Table 2). After adjusting for sex, age, and comorbidities, the risk of dementia was significant for patients with TTH than subjects without TTH (adjusted HR = 1.15, 95% CI = 1.05–1.27). Compared with patients aged 20–44 years, the risk of dementia was 14.5-times (95% CI = 9.93–21.1) and 105-times (95% CI = 72.2–152) higher in the age groups 45–64 and 65 years and older, respectively; therefore, the risk of dementia increased with age. In addition, the results of multivariate analyses showed patients with diabetes (adjusted HR = 1.41, 95% CI = 1.28–1.56), hypertension (adjusted HR = 1.52, 95% CI = 1.37–1.68), stroke (adjusted HR = 1.66, 95% CI = 1.44–1.91), depression (adjusted HR = 1.64, 95% CI = 1.43–1.88), head injury (adjusted HR = 1.52, 95% CI = 1.32–1.76), Parkinson’s disease (adjusted HR = 2.32, 95% CI = 1.91–2.81), migraine (adjusted HR = 1.23, 95% CI = 1.02–1.47), and COPD (adjusted HR = 1.29, 95% CI = 1.17–1.43) had a significantly higher risk of dementia than in those without counterpart comorbidity.

**Table 1:** Baseline demographic factors and comorbidity of study participants according to tension-type headache status.

	Non-TTH group N = 55632		TTH group N = 13908		p-value
Characteristics	n	%	n	%	
**Sex**					>0.99
Women	37096	66.7	9274	66.7	
Men	18536	33.3	4634	33.3	
**Age, years**					>0.99
20–44	23588	42.4	5897	42.4	
45–64	22008	39.6	5502	39.6	
≥ 65	10036	18.0	2509	18.0	
Mean (SD)^†^	48.8	(15.6)	49.1	(15.4)	0.06
**Comorbidity**					
Diabetes	4835	8.69	1447	10.4	<0.001
Dyslipidemia	9390	16.9	3665	26.4	<0.001
Hypertension	13932	25.0	5158	37.1	<0.001
IHD	7451	13.4	3178	22.9	<0.001
AF	376	0.68	112	0.81	0.11
HF	1309	2.35	512	3.68	<0.001
Stroke	1205	2.17	405	2.91	<0.001
Depression	1929	3.47	1552	11.2	<0.001
Head injury	2269	4.08	1136	8.17	<0.001
Parkinson’s disease	327	0.59	122	0.88	<0.001
Migraine	1181	2.12	1547	11.1	<0.001
COPD	4980	8.95	2061	14.8	<0.001

Abbreviation: TTH, tension-type headache; SD, standard deviation; COPD, chronic obstructive pulmonary disease; IHD, ischemic heart disease; AF, atrial fibrillation; HF, heart failure

^†^ Student’s t-test.

**Table 2:** Cox model measured hazard ratios and 95% confidence interval of dementia associated with tension-type headache and covariates.

			HR (95% CI)	
Characteristics	Event no.	IR	Univariate	Multivariate^†^
**Tension-type headache**				
No	1663	3.68	1.00	1.00
Yes	604	5.30	1.44 (1.31–1.58)***	1.15 (1.05–1.27)**
**Sex**				
Women	1419	3.69	1.00	1.00
Men	848	4.66	1.27 (1.17–1.39)***	0.99 (0.91–1.08)
**Age, years**				
20–44	29	0.12	1.00	1.00
45–64	491	2.14	18.5 (12.7–27.0)***	14.5 (9.93–21.1)***
≥ 65	1747	20.2	180 (125–260)***	105 (72.2–152)***
**Comorbidity**				
**Diabetes**				
No	1692	3.25	1.00	1.00
Yes	575	12.6	3.95 (3.59–4.34)***	1.41 (1.28–1.56)***
**Dyslipidemia**				
No	1372	2.95	1.00	1.00
Yes	895	8.84	3.03 (2.79–3.30)***	1.04 (0.95–1.14)
**Hypertension**				
No	674	1.60	1.00	1.00
Yes	1593	11.0	7.00 (6.40–7.66)***	1.52 (1.37–1.68)***
**IHD**				
No	1275	2.62	1.00	1.00
Yes	992	12.6	4.87 (4.48–5.29)***	1.10 (0.99–1.21)
**AF**				
No	2217	3.94	1.00	1.00
Yes	50	17.2	4.53 (3.42–5.99)***	0.98 (0.74–1.31)
**HF**				
No	2051	3.70	1.00	1.00
Yes	216	19.2	5.41 (4.70–6.23)***	1.07 (0.92–1.25)
**Stroke**				
No	2022	3.63	1.00	1.00
Yes	245	26.0	7.47 (6.54–8.53)***	1.66 (1.44–1.91)***
**Depression**				
No	2016	3.73	1.00	1.00
Yes	251	9.63	2.63 (2.31–3.00)***	1.64 (1.43–1.88)***
**Head injury**				
No	2063	3.82	1.00	1.00
Yes	204	8.02	2.14 (1.86–2.47)***	1.52 (1.32–1.76)***
**Parkinson’s disease**				
No	2154	3.82	1.00	1.00
Yes	113	43.3	11.7 (9.70–14.2)***	2.32 (1.91–2.81)***
**Migraine**				
No	2135	3.92	1.00	1.00
Yes	132	6.26	1.63 (1.36–1.94)***	1.23 (1.02–1.47)*
**COPD**				
No	1623	3.14	1.00	1.00
Yes	644	12.9	4.24 (3.87–4.64)***	1.29 (1.17–1.43)***

Abbreviation: IR, incidence density rates, per 1000 person-years; HR, hazard ratio; CI, confidence interval; TTH, tension-type headache; IHD, ischemic heart disease; AF, atrial fibrillation; HF, heart failure; COPD, chronic obstructive pulmonary disease.

^†^ Adjusted for sex, age, diabetes, dyslipidemia, hypertension, IHD, AF, HF, stroke, depression, head injury, Parkinson’s disease, migraine, and COPD in Cox proportional hazards regression.

* p<0.05,

** p<0.01,

*** p<0.001.

Sex-specific analysis showed that women with TTH had a greater risk of dementia than in those without TTH (adjusted HR = 1.25, 95% CI = 1.11–1.42; Table 3). Age-specific analysis showed that patients with TTH had a greatest magnitude risk of dementia compared with subjects without TTH in the age group of 65 years and older (adjusted HR = 1.13, 95% CI = 1.01–1.27). Comorbidity-specific analysis showed that patients with TTH had a significantly higher risk of dementia compared with subjects without TTH for study without any comorbidity (adjusted HR = 1.35, 95% CI = 1.22–1.49).

**Table 3:** Incidence density rates and hazard ratios of dementia according to tension-type headache status stratified by sex, age, and comorbidity.

	Tension-type headache						Compared to non-TTH group	
	No			Yes			HR (95% CI)	
Characteristics	Event no.	Person-years	IR	Event no.	Person-years	IR	Crude	Adjusted^‡^
**Sex**								
Women	1026	306954	3.34	393	77272	5.09	1.52 (1.35–1.71)***	1.25 (1.11–1.42)***
Men	637	145197	4.39	211	36745	5.74	1.31 (1.12–1.53)***	1.05 (0.89–1.24)
**Age, years**								
20–44	17	199546	0.09	12	50612	0.24	2.75 (1.31–5.76)**	1.60 (0.70–3.66)
45–64	345	183415	1.88	146	45979	3.18	1.69 (1.39–2.05)***	1.20 (0.98–1.47)
≥ 65	1301	69190	18.8	446	17425	25.6	1.36 (1.22–1.52)***	1.13 (1.01–1.27)*
**Comorbidity status**^†^								
No	276	273290	1.01	31	43434	0.71	0.70 (0.48–1.02)	1.05 (0.72–1.52)
Yes	1387	178861	7.75	573	70583	8.12	1.04 (0.95–1.15)	1.35 (1.22–1.49)***

Abbreviation: TTH, tension-type headache; IR, incidence density rates, per 1000 person-years; HR, hazard ratio; CI, confidence interval.

^†^ Patients with any one of diabetes, dyslipidemia, hypertension, IHD, AF, HF, stroke, depression, head injury, Parkinson’s disease, migraine, and COPD were classified as the comorbidity group.

^‡^ Model mutually adjusted for sex, age, and comorbidity.

* p<0.05,

** p<0.01,

*** p<0.001.

Furthermore, we examined the association between TTH and the risk of different subtypes of dementia, as shown in Table 4. We observed that patients with TTH had a null risk of Alzheimer’s disease and vascular dementia than those without TTH (adjusted HR = 1.24, 95% CI = 0.82–1.89 and adjusted HR = 0.93, 95% CI = 0.68–1.26, respectively). But, patients with TTH had a significantly increased risk of non-vascular dementia than those without TTH (adjusted HR = 1.21, 95% CI = 1.08–1.34). We further examined the associations between TTH and the risk of different subtypes of non-VD, as shown in S1 Table. The most common type of non-VD was uncomplicated senile dementia. Patients with TTH had a significantly higher risk of senile dementia with delusional or depressive features than those without TTH (adjusted HR = 1.72, 95% CI = 1.35–2.20). Only four participants had concomitant AD and VD.

**Table 4:** Incidence density rates and hazard ratios of dementia according to tension-type headache status.

	Tension-type headache				Compared to non-TTH group	
	No		Yes		HR (95% CI)	
Characteristics	Event no.	IR	Event no.	IR	Crude	Adjusted^†^
**Vascular dementia**	198	0.44	57	0.50	1.14 (0.85–1.53)	0.93 (0.68–1.26)
**Non-vascular dementia**	1465	3.24	547	4.80	1.48 (1.34–1.63)***	1.21 (1.09–1.34)***
Alzheimer’s disease	88	0.19	34	0.30	1.53 (1.03–2.28)*	1.24 (0.82–1.89)

ICD-9-CM: Alzheimer’s disease, 331.0; vascular dementia, 290.4.

Abbreviation: TTH, tension-type headache; IR, incidence density rates, per 1000 person-years; HR, hazard ratio; CI, confidence interval.

^†^ Model adjusted for sex, age, diabetes, dyslipidemia, hypertension, IHD, AF, HF, stroke, depression, head injury, Parkinson’s disease, migraine, and COPD in Cox proportional hazards regression.

* p<0.05,

*** p<0.001.

## Discussion

To the best of our knowledge, this is the first nationwide, population-based, longitudinal study to demonstrate an increased risk of dementia in TTH patients, which was 1.15-fold higher than in the non-TTH group, after adjustment for sex, age, and medical comorbidities. The incidence density rates of dementia increased with age and comorbidities for both TTH and non-TTH groups. Furthermore, TTH patients that were female, aged ≥65 years, or with medical comorbidities had a higher risk of subsequent dementia than patients with the same characteristics in the non-TTH group. Finally, the TTH group had a higher risk of developing non-VD than the non-TTH group.

The present results support those of previous studies reporting an association between TTH and dementia [3,22,24]. In a clinic-based, cross-sectional, observational study of the prevalence of headache in elderly subjects with various degrees of dementia, the most common type of headache was TTH, and there was a significant relationship between the degree of dementia and prevalence of headache [24]. More recent longitudinal, population-based, registry studies in Norway reported an association between non-migrainous headache and a higher risk of dementia, in addition to the relationship with migraine headache [3,22]. However, despite the larger sample size in these studies [3,22], subgroup analysis of non-migrainous headache, especially TTH, was not conducted. Furthermore, these studies failed to exclude some potential confounding medical factors. In this present study, we included a wide age range and considered possible medical and psychological confounding factors and comorbidities, providing results that are more generalizable to the general population.

The possible comorbidities of dementia, such as diabetes, dyslipidemia, hypertension, IHD, HF, stroke, depression, head injury, Parkinson’s disease, migraine, and COPD were more prevalent in the TTH group than in the non-TTH group in the present study; which is consistent that TTH might be associated with several psychiatric and medical conditions [25–27]. Nevertheless, despite a higher prevalence of comorbidities with TTH, the multivariate Cox analyses demonstrated that TTH was still an independent predictor for subsequent dementia risk.

In the multivariate analysis, patients with older age, diabetes, hypertension, stroke, depression, head injury, Parkinson’s disease, migraine, or COPD had significantly higher risks of dementia, after adjusting for covariates. Dementia is considered an age- or aging-related disorder [28], and dementia incidence increases with age in the general population. Similarly, the risk of dementia was higher in the 45–64-year-old and ≥65-year-old age groups than in the younger patients in the present study. However, there was no obvious sex-based difference in the occurrence of dementia in the present cohort (Table 2), which differs from previous studies reporting a higher prevalence of dementia among women than in men [29]. The heterogeneity in the study populations and differences in ethnicity, environmental factors, study designs, and outcome measurements might explain the different findings. However, the higher adjusted hazard ratios for dementia with diabetes, hypertension, stroke, depression, head injury, Parkinson’s disease, migraine, and COPD support the potential role of these conditions as risk factors for dementia [15–19,21,30].

In the dementia subgroup analysis, the incidence of dementia was higher in TTH patients with medical comorbidities than in patients with comorbidities but without TTH, reinforcing that TTH is a potential risk factor for dementia, especially in patients with dementia comorbidities. There was also an increased risk of developing dementia in female TTH patients. In addition to the higher risk of dementia for women in the general population [29], female TTH patients might experience more psychiatric disorders, such as major depression, than male TTH patients [31]. In the general population, women have a higher risk of depression than men; this could also be related with coexistent occult psychiatric disorders, as there are many other possible mechanisms for sex differences in risk.

We further examined the association between TTH and the risk of different subtypes of dementia. Compared with patients without TTH, TTH patients had a null risk of AD and VD, which is consistent with the lack of association between headache disorder and later development of AD in a previous study [22]. However, patients with TTH had a significantly increased risk of non-VD than those without TTH in the present study. Similarly, non-migrainous headache was reported at baseline more often among those who later developed dementia than in the reference group in another study [3]. However, these findings differ from those of a prior study that suggested migraine and non-migrainous headaches were associated with an increased risk of VD [22]. The reasons for the differences in results are unknown, although possible differences in ethnicity, environmental factors, methodological aspects, and clinical settings could contribute; furthermore, other degenerative brain conditions might contribute more to dementia in patients with TTH. Further research on TTH and non-VD should be done to provide more valuable insights into the relationship between these two conditions.

Several mechanisms might underlie the pathological association between TTH and dementia. First, THH is a common painful disorder. A previous meta-analysis of various pain disorders identified morphometric changes in the brain structures involved in pain processing (pain network), such as the thalamus, insular, prefrontal cortex, anterior cingulate, somatosensory cortex, basal ganglia, cerebellum, amygdala, hippocampus, and areas within the parietal and temporal cortices [32]. Interestingly, a large number of these structures are common to the following memory networks (memory recollection) that were identified in another meta-analysis: prefrontal, medial, and lateral temporal and anterior/retrosplenial/posterior cingulated cortices; temporoparietal junctions; insular; cerebellum; thalamus; and amygdalae [33]. Furthermore, in a previous structural neuroimaging study of chronic TTH, a decrease in gray matter was observed for most of these memory network structures, such as the cingulate cortex, insular, prefrontal area, and parahippocampus [34]. Therefore, marked changes in the overlapping pain and memory networks of the brain might explain the potential close interrelations between chronic pain and memory impairment in TTH patients. Second, subcortical white matter (WM) hyperintensity has been observed in patients with migraine, which could be related to cognitive decline in these patients [35,36]. Similarly, an increased risk of WM hyperintensity was reported in TTH patients [37], suggesting that this association is extends to non-migrainous headaches. WM hyperintensity has been associated with an increased risk of dementia [38]. Therefore, subtle brain WM changes might contribute to the increased risk of dementia in TTH patients. Finally, TTH is usually precipitated by the presence of stress and mental tension [1]. A previous large population-based study suggested an association between longstanding psychological stress in middle-aged women and the development of dementia later in life [39]. Although the underlying mechanism remained uncertain, the main hypotheses are related with the hypothalamic-pituitary-adrenal axis and the effects of glucocorticoids on the brain [40]. Therefore, stress, which is a potential trigger of TTH, may also potentially increase the risks of subsequent dementia. Future studies are needed to prove these speculations.

Our findings have several clinical implications. First, although the association between migraine and dementia has been controversial, the current results suggest that, in addition to migraine, TTH should be viewed as a potential risk factor for dementia. Owing to the risk for subsequent dementia, TTH patients should be evaluated for cognitive impairment both initially and at follow-up. Second, to aid in the early detection of dementia risk, clinicians should monitor TTH patients, particularly women, those aged ≥65 years, and those with medical comorbidities of dementia. Additionally, appropriate management of the related comorbidities might help to prevent or delay the onset of dementia.

The major strength of this study is the use of nationwide population-based data and the longitudinal, observational design with a lengthy follow-up; the large sample size provided sufficient statistical power to explore the relationship between TTH and dementia, while considering a variety of covariates. The rigorous diagnostic aspects are another strength; the diagnoses of TTH and dementia were based on the ICD-9 codes, as determined by qualified clinical physicians (not always neurologists) for the strictly audited reimbursement process. Furthermore, NHIRD covers a highly representative sample of Taiwan’s general population because the reimbursement policy is universal and operated by a single-buyer, the Taiwanese government. All insurance claims are scrutinized and coded by medical reimbursement specialists and peer reviewed according to the standard criteria for diagnoses in the study. Moreover, incorrect diagnoses or coding mistakes result in considerable penalties for the physicians. The reliability and validity of the NHI research database for epidemiologic investigations have been reported previously [41]. Therefore, the diagnoses and coding in this study should be highly reliable. Finally, the longitudinal design enabled assessment of the temporal relation between TTH and dementia, which could help determine a potential directional relationship.

However, there are certain limitations. First, the TTH cohort in the present study included only newly diagnosed TTH patients seeking medical help during the study period. Therefore, this group of TTH patients may not be fully representative of the general TTH population. Additionally, the clinical symptoms of TTH can be mild or infrequent; therefore, some patients with TTH might not have sought medical help and were not included in the TTH cohort. Collectively, it is possible that the risk of developing dementia for patients with TTH may have been underestimated. Second, information on potential confounders such as personal lifestyle (smoking status, alcohol consumption), body weight, body mass index, imaging results, and serum laboratory data was not available in the NHIRD. Third, TTH and dementia diagnoses were based on diagnostic codes that were entered by clinical physicians into the NHIRD database, rather than verified through face-to-face interviews with patients. Therefore, additional clinical information about TTH or dementia, such as the disease severity, duration, frequency of TTH, or stage of dementia was not available. Thus, our study was unable to differentiate between patients with episodic or chronic TTH or to determine the relationship of dementia stage with TTH characteristics. Fourth, the NHIRD dataset is derived from an administrative coding database that lacks detailed clinical data for dementia, such as predominant symptoms, mental or mood status, neuroimaging, and other laboratory results. Therefore, the precise diagnosis of other non-VD subtypes (excluding AD) was not available. This also made that the results regarding the non-VD subtypes should be interpreted with caution. Further research on the relationship between TTH and detailed non-VD subtypes are warranted. Finally, we could only identify a temporal association between TTH and dementia owing to the use of epidemiologic data from a large health care database; a direct causal relationship between these two disorders remains unclear and requires further research.

In conclusion, this large-scale, nationwide, population-based, longitudinal study demonstrated a temporal association between TTH and the risk of subsequent dementia, particularly in TTH patients who were female, aged ≥65 years, and with medical comorbidities. Although the exact mechanism is presently unclear, these findings might provide further insight into the association and possible shared pathophysiology between TTH and dementia. Dementia screening for TTH patients is necessary for early and effective interventions for both disorders. Additional studies are warranted to confirm an association and enrich our understanding of the interactions.

## Supporting Information

S1 Checklist The RECORD statement—checklist of items, extended from the STROBE statement, that should be reported in observational studies using routinely collected health data. (DOCX)

S1 Table Incidence density rates and hazard ratios for subtypes of non-vascular dementia according to tension-type headache status. Abbreviation: TTH, tension-type headache; IR, incidence density rates, per 1000 person-years; HR, hazard ratio; CI, confidence interval. † Model adjusted for sex, age, diabetes, dyslipidemia, hypertension, IHD, AF, HF, stroke, depression, head injury, Parkinson’s disease, migraine, and COPD in Cox proportional hazards regression. * p<0.05, *** p<0.001.
(DOCX)

## Abbreviations

CIs: confidence intervals
HRs: hazard ratios
ICD-9-CM: International Classification of Diseases, Ninth Revision, Clinical Modification
NHIRD: National Health Insurance Research Database
VD: vascular dementia
TTH: tension-type headache
