# Supplementary material for: Increased Risk of Dementia in Patients with Tension-Type Headache: A Nationwide Retrospective Population-Based Cohort Study
Source: PLoS One. 2016 Jun 7;11(6):e0156097. doi: 10.1371/journal.pone.0156097 (PMC4896423; doi:10.1371/journal.pone.0156097)
Supplement: S1 Table — Abbreviation: TTH, tension-type headache; IR, incidence density rates, per 1000 person-years; HR, hazard ratio; CI, confidence interval. † Model adjusted for sex, age, diabetes, dyslipidemia, hypertension, IHD, AF, HF, stroke, depression, head injury, Parkinson’s disease, migraine, and COPD in Cox proportional hazards regression. * p<0.05, *** p<0.001. (DOCX) [file pone.0156097.s002.docx]

Supplementary Table 1. Incidence density rates and hazard ratios for subtypes of non-vascular dementia according to tension-type headache status

|  |  | |  | | **Compared to non-TTH group** | |
| --- | --- | --- | --- | --- | --- | --- |
|  | **Non-TTH group** | | **TTH group** | | **HR (95% CI)** | |
| **Sub-group** | **Event no.** | **IR** | **Event no.** | **IR** | **Crude** | **Adjusted^†^** |
| **Non-vascular dementia** | 1465 | 3.24 | 547 | 4.80 | 1.48 (1.34-1.63)*** | 1.21 (1.09-1.34)*** |
| Alzheimer’s disease (331.0) | 88 | 0.19 | 34 | 0.30 | 1.53 (1.03-2.28)* | 1.24 (0.82-1.89) |
| Senile dementia, uncomplicated (290 and 290.0) | 780 | 1.73 | 256 | 2.25 | 1.30 (1.13-1.50)*** | 1.08 (0.93-1.25) |
| Presenile dementia (290.1) | 146 | 0.32 | 54 | 0.47 | 1.46 (1.07-2.00)* | 1.09 (0.78-1.51) |
| Senile dementia with delusional or depressive features (290.2) | 207 | 0.46 | 108 | 0.95 | 2.07 (1.64-2.61)*** | 1.72 (1.35-2.20)*** |
| Senile dementia with delirium (290.3) | 51 | 0.11 | 16 | 0.14 | 1.24 (0.71-2.18) | 0.98 (0.54-1.77) |
| Dementia in conditions classified elsewhere (290.8, 290.9, and 294.1) | 193 | 0.43 | 79 | 0.69 | 1.62 (1.25-2.11)*** | 1.31 (0.99-1.72) |

Abbreviation: TTH, tension-type headache; IR, incidence density rates, per 1000 person-years; HR, hazard ratio; CI, confidence interval.

^†^ Model adjusted for sex, age, diabetes, dyslipidemia, hypertension, IHD, AF, HF, stroke, depression, head injury, Parkinson’s disease, migraine, and COPD in Cox proportional hazards regression.

* p<0.05, *** p<0.001.
